# Peroneus Tertius Tendon Tear: A Rare Cause of Lateral Ankle Pain

**DOI:** 10.7759/cureus.577

**Published:** 2016-04-19

**Authors:** Edward Derrick, Miguel Flores, Kurt Scherer, Laura Bancroft

**Affiliations:** 1 Diagnostic Radiology, Florida Hospital-Orlando

**Keywords:** peroneal tendons, mri, mri musckuloskeletal, musculoskeletal injuries, muscoloskeletal, ankle, foot ankle, fibularis tertius, peroneus tertius

## Abstract

The peroneus tertius (PT) muscle is a variably present muscle, uncommonly found in humans. Injury to the PT tendon is rare with virtually no cases reported in the literature. As a consequence of the rarity of this injury, there is little clinical information regarding injury or rupture of the PT muscle and tendon. We present a case of injury involving this rare anatomical variant. Magnetic resonance (MR) imaging demonstrates a short segment longitudinal split tear adjacent to the tendinous insertion of the peroneus tertius muscle. Knowledge of this rare anatomic variant and the potential for associated pathology is critical in the management of the patient. Directing the orthopedic surgeon, or podiatrist, to this finding is critical for directing intervention.

## Introduction

The peroneus tertius (PT) muscle, also referred to as the fibularis tertius muscle, is a small muscle of the lower extremity whose principal action is weak dorsiflexion and eversion of the foot [1]. Additionally, the PT muscle counters the inverting force of the tibialis anterior, effectively leveling the foot. As such, it is thought that the PT muscle played a role in the evolution of bipedal gait; it is predominantly present in humans, and is often absent among other primates [2]. We present a previously unreported high-grade tear injury of the peroneus tertius tendon with associated injuries involving the tendinous insertions of the peroneus longus and peroneus brevis. Informed consent from the patient was waived for this study.

## Case presentation

A 39-year-old female presented with pain, burning sensation, and swelling of the left ankle requiring evaluation for a suspected anterior talofibular ligament tear and an osteochondral defect. MRI examination revealed the patient to have a peroneus tertius and quartus, both uncommon anatomic variants. Moreover, a rare high-grade tear injury of the peroneus tertius tendon was identified, which has not been previously reported in the literature.

**Figure 1:**
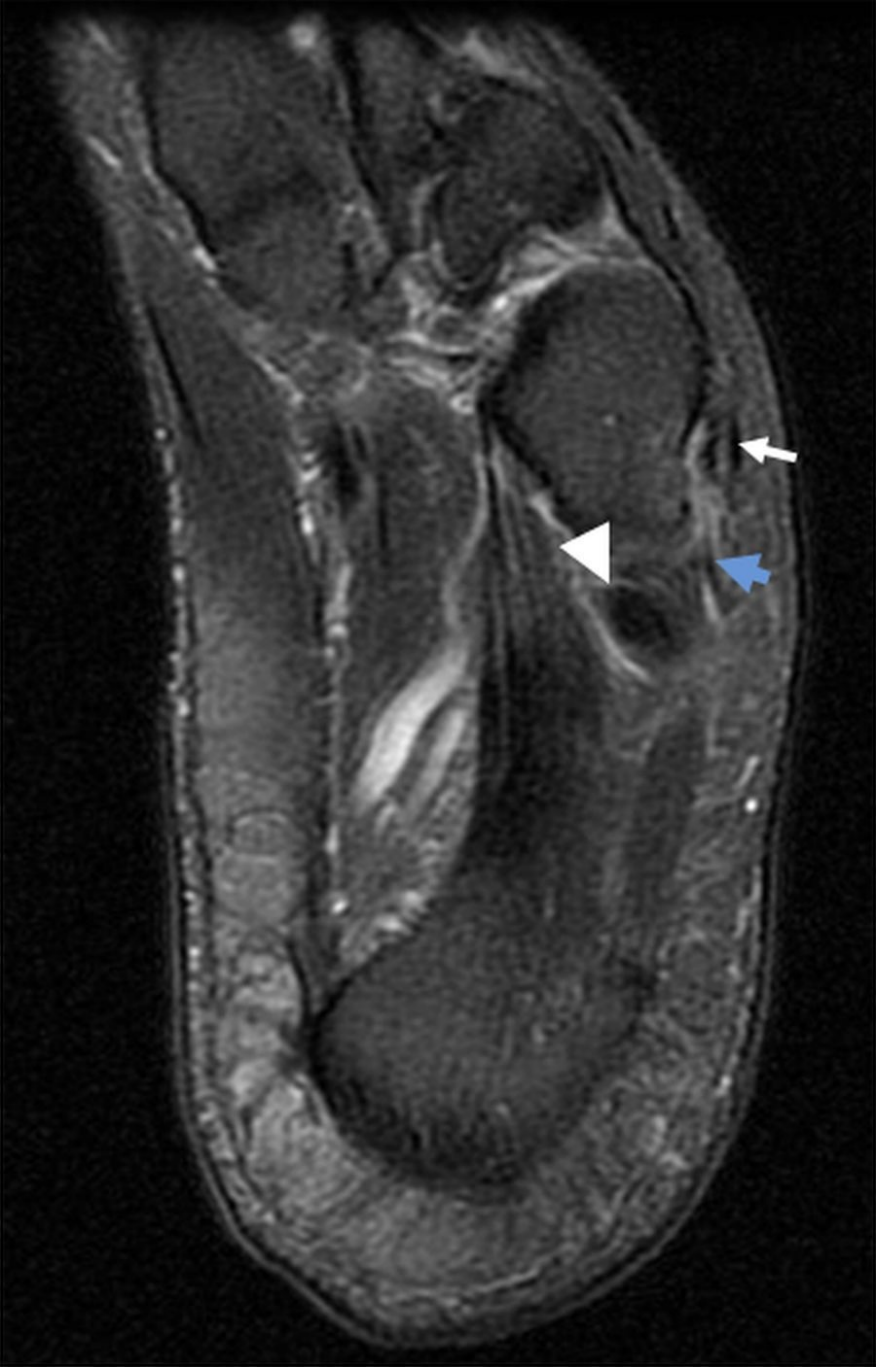
Longitudinal Split Tears of the Peroneus Tertius, Longus and Brevis Tendons T2 prolongation along the peroneus tertius tendon sheath is present, with a short segment longitudinal split tear immediately adjacent to its insertion at this level (white arrow). There is an additional high-grade longitudinal split tear of the peroneus longus tendon (arrowhead). A complex longitudinal split tear of the peroneus brevis tendon was also present, with non-visualization of its distal insertional fibers presumably related to proximal retraction of the tendon fibers (blue arrow).

Increased signal on T2-weighted images was demonstrated in the peroneus tertius tendon sheath with a short segment longitudinal split tear immediately adjacent to its insertion. Additionally there was a high-grade, longitudinal split tear of the peroneus longus tendon and a complex longitudinal split tear of the peroneus brevis tendon (Figure 1).

**Figure 2:**
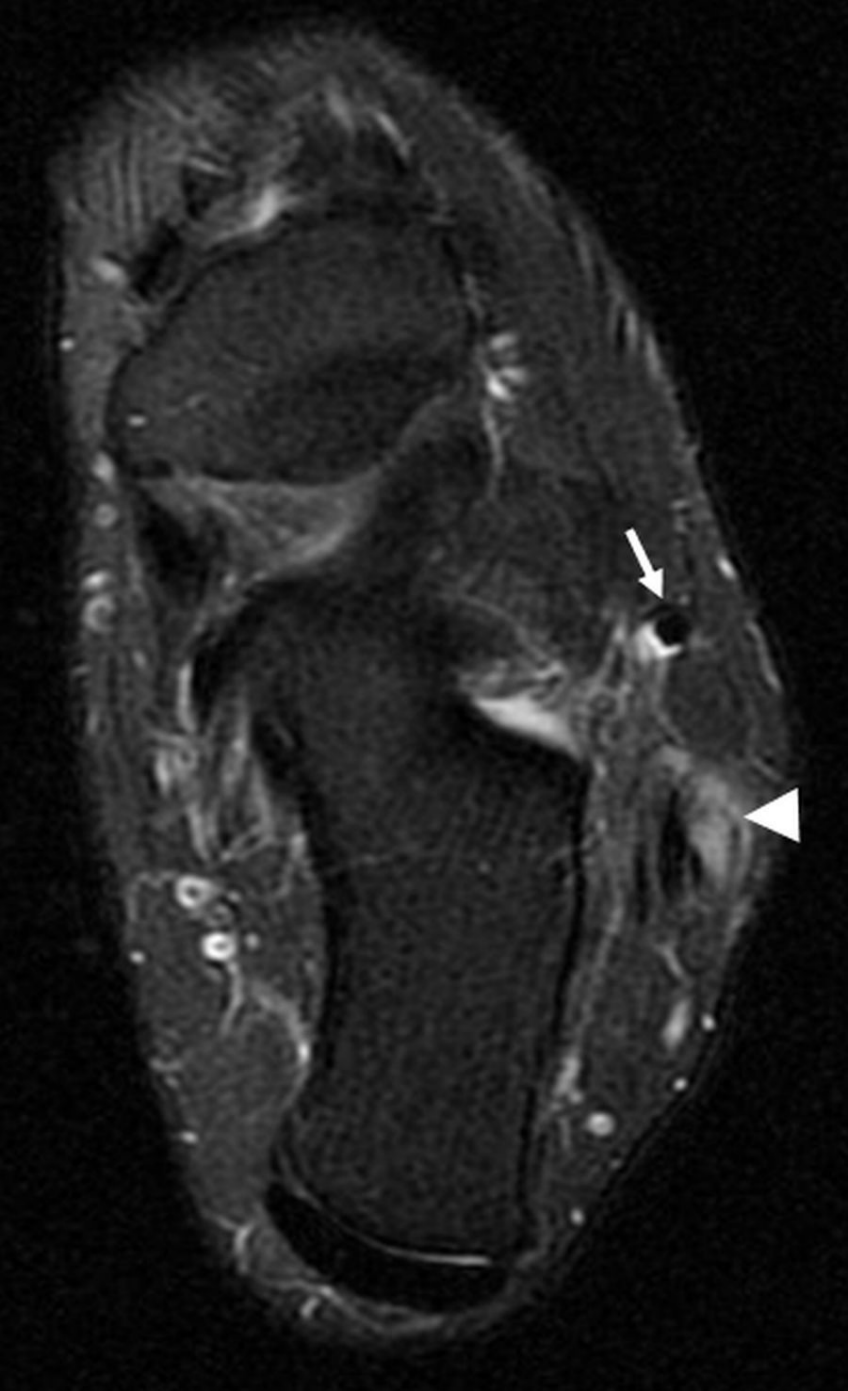
Tenosynovitis of the Common Peroneal Tendon Sheath and Peroneus Tertius Axial T2-weighted fat suppressed image inferior to the lateral malleolus shows T2-hyperintensity surrounding both the common peroneal tendon sheath (arrowhead) and the peroneus tertius (arrow), consistent with tenosynovitis.

**Figure 3:**
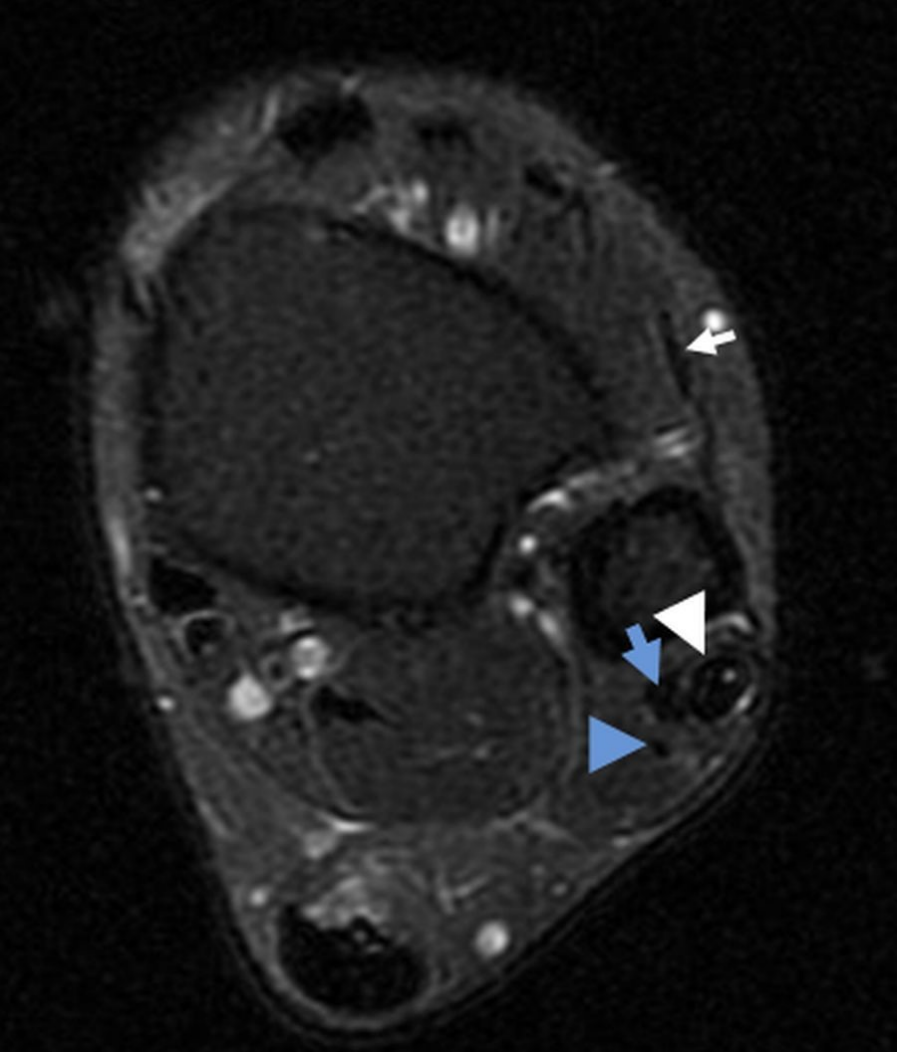
Peroneus Tertius and Quartus Axial T2-weighted image superior to the lateral malleolus demonstrates a normal peroneus tertius tendon (white arrow) anterior to the fibula. The peroneus longus tendon (white arrowhead) contains small foci of internal hyperintense signal, whereas the peroneus brevis (blue arrow) and quartus (blue arrowhead) tendons are normal in signal intensity.

T2 sequences also demonstrated elevated signal around the common peroneal tendon sheath, consistent with tenosynovitis (Figure 2). Of note, there was an accessory peroneus quartus muscle (Figure 3).

## Discussion

The PT muscle originates at the anterior distal third of the fibula and courses beneath the superior extensor retinaculum before entering the tendinous sheath of the tibialis anterior. The peroneus tertius tendon subsequently passes beneath the inferior extensor retinaculum and inserts on the dorsal base of the fifth metatarsal. Several reports in the literature support morphological variation of the peroneus tertius, including variation in size, site, number of origins, and multiple points of insertion [2-6]. The prevalence of the PT muscle ranges from 49% [7] to 94% [8] in anatomic studies by Ramirez and Rourke, and congenital absence of the muscle has not been associated with an increased risk of ankle ligamentous injury [9].

Injury to the peroneus tertius tendon is rare, with no known cases reported in the literature. Therefore, there is little information pertaining to the clinical significance, diagnostic maneuvers, and therapeutic options for patients presenting with PT injury or rupture. In this case report, MRI sequences demonstrated a short segment, longitudinal split tear near the insertion of the peroneus tertius tendon, as well as tenosynovitis (Figure 1). There were also longitudinal split tears of both the peroneus longus and peroneus brevis tendons (Figure 1).

The peroneus quartus muscle was also present in our case (Figure 3). The peroneus quartus is a muscle that typically arises from the peroneus brevis and attaches to the calcaneus and has been used for surgical reconstruction of the retromalleolar groove, functioning as a strap to stabilize the peroneal tendons. The peroneus quartus is estimated to have a prevalence of approximately 6.6 to 13% and has been associated with pain and weakness of the ankle. There is evidence to suggest a predisposition to developing longitudinal tears in the tendon of the peroneus brevis, possibly pertaining to laxity in the superior peroneal retinaculum and a prominent retrotrochlear eminence [10].

## Conclusions

Injury to the peroneus tertius tendon is rare, with no previously reported cases in the literature. Therefore, there is little information pertaining to the clinical significance, diagnostic maneuvers, and therapeutic options for patients presenting with PT injury or rupture. Knowledge of this rare anatomic variant and the potential for associated pathology is critical in the management of patients who present with injury involving this uncommon variant.

## References

[1] Jungers WL, Meldrum DJ, Stern JT (1993). The functional and evolutionary significance of the human peroneus tertius muscle. J Hum Evol.

[2] Joshi SD, Joshi SS, Athavale SA (2006). Morphology of peroneus tertius muscle. Clin Anatomy.

[3] Mehta V, Gupta V, Nayyar A (2011). Accessory muscle belly of peroneus tertius in the leg--a rare anatomical variation with clinical relevance--utility in reconstructions. Morphologie.

[4] Yildiz S, Yalcin B (2012). An unique variation of the peroneus tertius muscle. Surg Radiol Anat.

[5] Sirasanagnadla SR, Bhat KM, Nayak SB, Shetty P, Thangarajan R (2014). A rare case of variant morphology of peroneus tertius muscle. J Clin Diagn Res.

[6] Jana R, Roy TS (2011). Variant insertion of the fibularis tertius muscle is an evidence of the progressive evolutionary adaptation for the bipedal gait. Clin Practice.

[7] Ramirez D, Gajardo C, Caballero P, Zavando D, Cantín M, Suazo G (2010). Clinical evaluation of fibularis tertius muscle prevalence. Int J Morphol.

[8] Rourke K, Dafydd H, Parkin IG (2007). Fibularis tertius: revisiting the anatomy. Clin Anat.

[9] Witvrouw E, Borre KV, Willems TM, Huysmans J, Broos E, De Clercq D (2006). The significance of peroneus tertius muscle in ankle injuries: a prospective study. Am J Sports Med.

[10] Zammit J, Singh D (2003). The peroneus quartus muscle. Anatomy and clinical relevance. Bone Joint J.

